# Metagenomic search of viral coinfections in herpes simplex encephalitis patients

**DOI:** 10.1007/s13365-023-01157-9

**Published:** 2023-07-25

**Authors:** Karol Perlejewski, Marek Radkowski, Małgorzata Rydzanicz, Tomasz Dzieciątkowski, Steffi Silling, Magdalena Wieczorek, Michał Makowiecki, Andrzej Horban, Tomasz Laskus

**Affiliations:** 1 Department of Immunopathology of Infectious and Parasitic Diseases, Medical University of Warsaw, Pawinskiego 3c, 02-106 Warsaw, Poland; 2 Department of Medical Genetics, Medical University of Warsaw, Pawinskiego 3c, 02-106 Warsaw, Poland; 3 Department of Microbiology, Medical University of Warsaw, Chalubińskiego 5, 02-004 Warsaw, Poland; 4 Institute of Virology, National Reference Center for Papilloma- and Polyomaviruses, University of Cologne, Faculty of Medicine, University Hospital Cologne, Fürst-Pückler-Straße 56, 50935 Cologne, Germany; 5 Department of Virology, National Institute of Public Health-National Institute of Hygiene, Chocimska 24, 00-791 Warsaw, Poland; 6 Department of Adults Infectious Diseases, Medical University of Warsaw, Wolska 37, 01-201 Warsaw, Poland

**Keywords:** Viral coinfection, Central nervous system, Metagenomics, Encephalitis

## Abstract

Little is known about concomitant central nervous system (CNS) infections by more than one virus. Current diagnostics are based on molecular tests for particular pathogens making it difficult to identify multi-viral infections. In the present study, we applied DNA- and RNA-based next-generation sequencing metagenomics (mNGS) to detect viruses in cerebrospinal fluids from 20 patients with herpes simplex encephalitis. Coinfection was detected in one patient: sequences in cerebrospinal fluids matched enterovirus A (2.660 reads; 4% of recovered genome) and enterovirus B (1.571 reads; 13% of recovered genome). Subsequent PCR combined with serotyping allowed to identify human echovirus 6, a representative of enterovirus B. Several other mNGS hits (human pegivirus, Merkel cell polyomavirus, human papillomavirus type 5) were not considered to represent a genuine signal as they could not be confirmed by specific RT-PCR/PCR. HSV DNA, while being detectable by PCR in every patient, was detected by mNGS in only one. In conclusion, contaminations and false signals may complicate mNGS interpretation; however, the method can be useful in diagnostics of viral coinfections in CNS, particularly in the case of rare pathogens.

## Introduction

Encephalitis is an inflammatory process of brain parenchyma and is often associated with high mortality and long-term neurological sequelae (Bookstaver et al. 2017). It could be triggered by autoimmune mechanisms but is most often caused by infectious agents (Tunkel et al. 2008) among which viruses predominate constituting between 50 and 69% of all cases (Glaser et al. 2006; Kupila et al. 2006). More than a hundred viral species were identified so far as possible causative agents, but *Herpesviridae*, *Picornaviridae*, and arboviruses are the most commonly encountered in Europe and North America (Hinson and Tyor 2001; Salimi et al. 2016).

Fast and accurate identification of causative pathogens is often crucial for the timely implementation of proper treatment of encephalitis (Venkatesan and Geocadin 2014). Current diagnostics are largely based on serology and/or detection of viral genetic material by PCR/RT-PCR in cerebrospinal fluid (CSF); (Kennedy 2004). However, due to large number of potential viral agents, typical low CSF viral loads and high costs of tests which limit the diagnostics to pathogens that are most relevant for the particular epidemiological setting in as many as 60% of encephalitis cases the causative agent remains unidentified (Chaudhuri and Kennedy 2002; Kennedy et al. 2017). These limitations of routine diagnostics particularly affect the identification of rare and emerging pathogens and multi-viral infections (Radmard et al. 2019). Some studies reported finding more than one pathogen in CSF from encephalitis patients (Rasti et al. 2016; Weinberg et al. 2005) and in two unusual cases as many as six different viruses were detected (Kumar et al. 2019, 2020). It was postulated that viral coinfection could modify the course of encephalitis and disease severity (Kumar et al. 2020).

Next-generation sequencing-based metagenomics (mNGS) holds the promise to remedy the problem of identification of several pathogens simultaneously, and it has been already successfully applied in the setting of encephalitis (Perlejewski et al. 2015; Tan le et al. 2013; Wilson et al. 2014). In the current study, we employed mNGS protocols to analyze CSF collected from 20 patients with confirmed diagnosis of herpes simplex encephalitis (HSE) to search for confecting viral pathogens. HSE patients were chosen as the study group since human herpes simplex virus type 1 (HSV1) is the most frequent cause of viral encephalitis (24%) in Poland (Popiel et al. 2017).

## Methods

### Patients

We analyzed adult (≥ 18 yrs.) patients with HSE, who were hospitalized in the Municipal Hospital for Infectious Diseases in Warsaw. Inclusion criteria were HSV1 detection in the CSF by a commercial assays and availability of CSF sample for the current analysis. Encephalitis was defined as an acute onset of illness with altered mental status or decreased level of consciousness or seizures or focal neurological signs together with at least one abnormality of the cerebrospinal including white blood cell count ≥ 4 cells/mm^2^ or protein level ≥ 40 mg/dl (Popiel et al. 2017). Twenty patients (15 men, 5 women, median age 36 years; range 20 to 79 years) fulfilled these criteria and were the subjects of the study. Written informed consent was obtained from patients or from their close relatives if the patient was unable to give consent because of his condition. Consent had to be confirmed once patient’s condition improved. The study was approved by the Internal Review Board of the Medical University of Warsaw.

A lumbar puncture was performed in all patients at admission. CSF samples were centrifuged (at 1200 rpm for 20 min at 4 °C), aliquoted and kept frozen at − 80 °C until current analysis.

The most commonly reported symptoms were fever (50%), headache (50%) and altered state of consciousness (45%). The average total cell count in CSF was 266 cells/µl with mean concentration of 0.6 g/l for protein and 3.49 mmol/l for glucose. Basic epidemiological and clinical information on the 20 HSE patients is provided in Table 1.

**Table 1:** Clinical and laboratory (*nd*, no data/not done; *Altered con.*, altered state of consciousness; *Prot*, protein; *LDH*, lactate dehydrogenase) characteristics of 20 patients with herpes simplex encephalitis (HSE)

				**Clinical presentation**				**Cerebrospinal fluid (CSF) analysis**				
	**HSV viral** **load** **in CSF** **(copies/** **ml.)**	**Age (yrs.)**	**Sex**	**Fever (±)**	**Headache (±)**	**Altered** **con.** **(±)**	**Seizures** **(±)**	**Cytosis (in 1 μl)**	**% of lymph.**	**Prot. (g/l)**	**LDH (mmol/l)**	**Glucose (mmol/l)**
**Pt.1**	120	67	F	+	-	+	-	2453	nd	1.08	nd	0.6
**Pt.2**	160	47	M	-	-	-	+	1	nd	0.28	1.42	3.79
**Pt.3**	148	36	M	-	-	+	-	3	nd	0.20	1.30	3.71
**Pt.4**	230	25	M	-	-	+	-	2	nd	0.39	1.25	4.28
**Pt.5**	129	20	M	+	+	-	-	723	75	0.32	1.63	4.94
**Pt.6**	310	38	M	+	+	-	-	96	87	0.16	1.37	3.05
**Pt.7**	344	38	F	-	-	-	-	17	74	0.33	1.47	3.47
**Pt.8**	152	28	M	+	+	+	-	15	72	0.71	2.17	5.81
**Pt.9**	76	38	M	+	+	-	-	182	75	0.83	1.40	3.26
**Pt.10**	72	80	M	-	-	+	-	1	nd	0.57	2.01	5.03
**Pt.11**	72	64	F	-	-	-	-	13	nd	1.02	1.62	2.48
**Pt.12**	76	36	M	+	-	+	-	1225	43	1.78	3.94	1.26
**Pt.13**	246	27	M	-	+	-	-	79	nd	0.41	1.14	2.27
**Pt.14**	82	54	M	+	-	+	-	3	nd	0.62	1.24	3.16
**Pt.15**	76	34	F	+	+	-	+	25	nd	0.39	1.4	3.8
**Pt.16**	74	62	M	+	-	-	+	28	nd	0.87	1.54	4.5
**Pt.17**	150	79	F	+	+	+	-	43	nd	0.84	2.42	3.41
**Pt.18**	60	27	M	-	-	+	+	2	nd	0.19	1.23	2.29
**Pt.19**	76	32	M	-	+	-	+	83	nd	0.5	1.76	4.3
**Pt.20**	144	59	M	-	-	-	+	1	nd	0.53	2.06	3.82

The previous HSE diagnosis was confirmed by HSV1-DNA detection in CSF by in-house PCR (Machura et al. 2015). HSV1 viral loads in CSFs ranged from 60 to 344 copies/per ml. All patients were negative for tick-borne encephalitis virus (TBEV) and *Borrelia* spp. infection based on routine serological testing using Serion ELISA classic TBEV IgG/IgM (Institut Virion/Serion GmbH, Würzburg, Germany) and recomLine Borrelia IgM/IgG (Mikrogen Diagnostik, Germany), respectively.

### Metagenomic next-generation sequencing

 The mNGS RNA and DNA analysis were performed as described previously (Perlejewski et al. 2020). In short, 225 µl of CSF was filtered (Millex-HV Syringe Filter Unit; pore size 0.45 μm; Merck KgaA, Germany) and digested with 2U of TURBO DNase (Thermo Fisher Scientific, USA) for 30 min. Next, RNA and DNA were extracted with TRIzol LS (Thermo Fisher Scientific, USA) and NucleoSpin Plasma XS kit (Macherey–Nagel, Germany), respectively. Extracted RNA was suspended in 5 μl and DNA was eluted in 12 μl of water.

Due to low yields of DNA/RNA which is common for CSF extraction, a preamplification step was employed to generate sufficiently large NGS libraries for sequencing. RNA and DNA were preamplified by a single-primer isothermal amplification (Ribo-SPIA) using Ovation RNA-Seq V2 system (NuGEN, San Carlos, USA) and SeqPlex Enhanced DNA Amplification kit (Sigma-Aldrich, USA), respectively. Preamplified cDNA/DNA was purified using Agencourt AMPure XP beads (Beckman Coulter, USA); (0.8 ratio to reaction mixture) and eluted in 30 μl of water.

 NGS libraries were prepared with Nextera XT Kit (Illumina, USA) using 1 ng of preamplified cDNA/DNA and employing 14 cycles during indexing and performing final purification with 0.6 ratio of Agencourt AMPure XP beads (Beckman Coulter, USA). Quality of NGS libraries was evaluated using Bioanalyzer (Agilent Technologies, USA), and the double-indexed DNA was measured with Qubit dsDNA HS kit (Thermo Fisher Scientific, USA). Sequencing was performed on HiSeq 1500 System (Illumina, USA) generating 101nt paired-end reads.

#### Data analysis

After sequencing reads were evaluated for their quality with FastQC software, then filtered and trimmed using BBmap and Trimmomatic, respectively (Bolger et al. 2014). Filtered reads were mapped to human reference genome (GRCh38, GenBank) using Stampy (Lunter and Goodson 2011). Remaining non-human reads were aligned by Bowtie2 (Langmead and Salzberg 2012) to viral reference genomes obtained from NCBI Reference Sequence Database (RefSeq). Viral reads were sorted and counted using SAMtools (Li et al. 2009) and phyloseq (McMurdie and Holmes 2013) package in R. Genome assembly was performed using SPAdes (Bankevich et al. 2012). Coverage rates and visualization of viral alignments were performed using CLC Genomics Workbench (Qiagen, USA).

For positive mNGS virus detection at least two unique, non-overlapping reads mapping to a particular virus had to be identified and similar criteria have been previously used by others (Kufner et al. 2019; Schlaberg et al. 2017). Each positive mNGS result had to be confirmed by specific PCR/RT-PCR.

### Specific PCR/RT-PCRs

RNA and DNA were extracted from 200 µl of CSF using Trizol LS (Thermo Fisher Scientific, USA) and NucleoSpin Plasma XS kit (Macherey Nagel, Germany), respectively. All samples were tested for a HSV-1 using in-house quantitative PCR. Furthermore confirmatory PCRs were performed for the following viruses identified by mNGS: enteroviruses (assays detecting EV: Coxsackie A9, A16, B2, B3, B4, B5; ECHO 5, 6, 9, 11, 18, 30, and EV 71); (Les et al. 2010), HPgV (Radkowski et al. 1999), and MCPyV (Katano et al. 2009). To verify the presence of *Papillomaviridae* specific PCRs detecting alpha (Schulze et al. 2016) and beta (Berkhout et al. 1995) HPVs were used.

### Enterovirus serotyping

Enterovirus strain identified by amplification was further isolated from CSF using RD (rhabdomyosarcoma) cell line according to the standard procedure recommended by the World Health Organization (WHO 2004). RD cells were cultivated in minimal essential medium (MEM) supplemented with 10% fetal bovine serum. The identification of the isolate was performed by sequencing of the VP1 coding gene.

Viral RNA was extracted from cell culture supernatant using QIAamp Viral RNA Mini Kit (Qiagen, USA). Extracted RNA was amplified by a combined RT and first round PCR using Superscript III (Invitrogen, USA) followed by a second amplification reaction with nested primers for enteroviral species A and B VP1 as described previously (Leitch et al. 2009). The resulting DNA templates were processed in cycle sequencing reaction with BigDye 3.1. The product of sequencing was run in an automated genetic analyzer (Applied Biosystems, USA). Sequences were manually edited using BioEdit program and examined in terms of closest homologue sequence using BLAST software.

## Results

Metagenomic analysis was performed in all 20 HSE patients with the exception of Pt.7 in whom only RNA-based mNGS was done due to the limited amount of available CSF. Altogether 39 NGS libraries were sequenced generating totally 505,023,484 reads with an average number of 12,949,320 reads per sample. Non-human reads were filtered after alignment to GRCh38, and their number ranged from 39,916 to 13,641,768 with more of non-human sequences found in RNA-based than in DNA-based mNGS analysis (7,070,395 reads vs. 1,898,390 reads). The highest number of viral reads was present in RNA-mNGS of Pt.19 (299,583 reads, 2.334% of total reads), whereas the lowest in DNA-mNGS of Pt.16 (32 reads; 0.003% of total reads). The majority (77.52%) of detected viral sequences aligned to bacteriophage genomes. Sequences common for our lab contamination background which were identified in various previous mNGS runs were removed (Bukowska-Osko et al. 2016; Perlejewski et al. 2015, 2020). Only animal viruses were considered as possible etiologic agents of encephalitis. Results of mNGS together with mapping information are shown in Table 2.

**Table 2:** Results of mNGS analysis in cerebrospinal fluid from 20 patients with herpes simplex encephalitis (HSE). Only animal viruses represented by at least two unique, non-overlapping reads mapping to a particular virus were included

**Sample**	**DNA/RNA**	**Raw reads**	**Nonhuman reads**	**Viral reads**	**% of viral reads**	**% of phages**	**Identified eukaryotic viruses in mNGS** (number of reads)
**PT.1**	DNA	8738619	1970775	2761	0.0316	94.60	-
	RNA	11164300	10159043	4378	0.0392	67.06	-
**PT.2**	DNA	10191887	3864175	4138	0.0406	78.61	-
	RNA	8725176	8100284	4196	0.0481	80.62	-
**PT.3**	DNA	13888334	989149	734	0.0053	90.60	-
	RNA	10380433	7721722	513	0.0049	54.39	-
**PT.4**	DNA	14167138	919554	1007	0.0071	96.43	Human papillomoavirus type 5 (12)
	RNA	13923233	11922594	1532	0.0110	60.44	-
**PT.5**	DNA	24267354	13641768	12,897	0.0531	99.29	Human papillomavirus type 5 (51) Colobus guereza papillomavirus 2 (6)
	RNA	21958931	12010837	6284	0.0286	98.76	Human enterovirus B (1571) Human enterovirus A (2660)
**PT.6**	DNA	14815771	49304	60	0.0004	53.33	-
	RNA	12281286	9342932	865	0.0070	60.12	-
**PT.7**	RNA	14216204	10953046	3812	0.0268	78.78	-
**PT.8**	DNA	22055534	632589	1295	0.0059	81.54	-
	RNA	12011637	4720340	3324	0.0277	87.58	-
**PT.9**	DNA	11747029	118128	1283	0.0109	93.69	-
	RNA	9617944	8469304	6679	0.0694	40.17	-
**PT.10**	DNA	11779519	1972921	4881	0.0414	74.80	Merkel cell polyomavirus (12)
	RNA	10447083	2671749	2493	0.0239	66.23	-
**PT.11**	DNA	11808555	4565386	5344	0.0453	53.84	-
	RNA	23166447	2665196	4807	0.0207	76.80	-
**PT.12**	DNA	11710926	534221	818	0.0070	95.84	-
	RNA	11350922	2037914	2185	0.0192	82.79	-
**PT.13**	DNA	10959230	979838	478	0.0044	57.32	-
	RNA	13164677	2255501	3641	0.0277	77.81	-
**PT.14**	DNA	13072234	2683022	2325	0.0178	62.62	-
	RNA	11126665	7591552	5738	0.0516	99.86	Human pegivirus (4)
**PT.15**	DNA	10804105	526786	838	0.0078	82.58	-
	RNA	11939100	7315710	1238	0.0104	91.68	-
**PT.16**	DNA	11417798	39916	32	0.0003	62.50	-
	RNA	13234600	2116512	1654	0.0125	91.90	-
**PT.17**	DNA	5307348	169597	263	0.0050	53.61	-
	RNA	10855104	6384650	2317	0.0213	40.66	-
**PT.18**	DNA	13643662	1073405	1092	0.0080	85.53	-
	RNA	14522750	11165348	13,367	0.0920	76.79	Human herpesvirus 1 (2)
**PT.19**	DNA	14156833	1325881	1951	0.0138	94.31	-
	RNA	12835468	8011157	299,583	2.3340	99.66	-
**PT.20**	DNA	9423656	85381	34	0.0004	97.06	-
	RNA	14145992	8881162	4072	0.0288	83.06	-

**Fig. 1:**
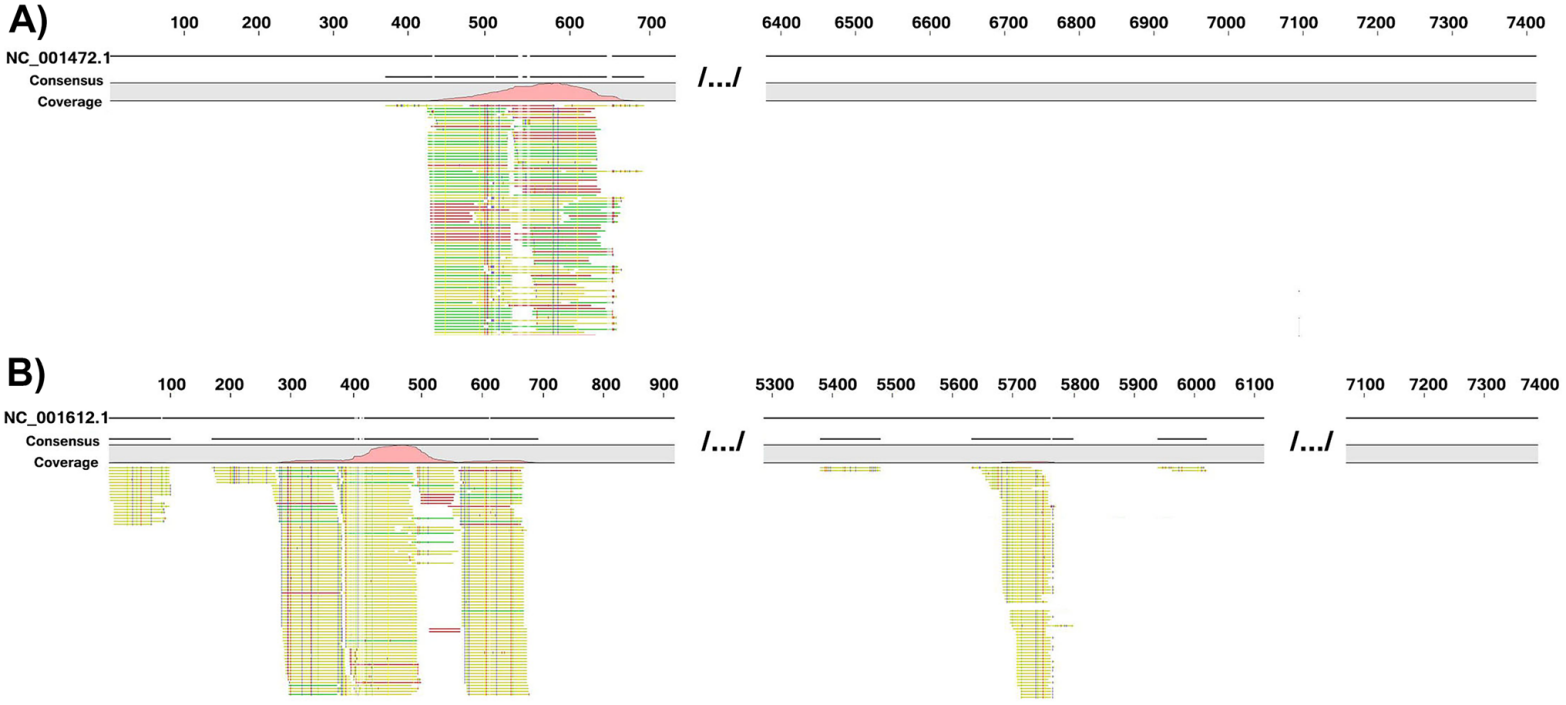
Identification of EV-RNA by mNGS in cerebrospinal fluid of Pt.5. **A** Sequence alignment (4% of recovered genome) to enterovirus A genome (NC_001612.1). **B** Sequence alignment (13% of recovered genome) to enterovirus B genome (NC_001472.1)

In CSF of Pt.5 mNGS we identified sequences specific for enterovirus A (EV A) and B (EV B). Despite higher number of reads (2.660) aligning to EV A than to EV B (1.571) more EV B (13%), genome was recovered than EV A (4%) (Fig. 1).

**Fig. 2:**
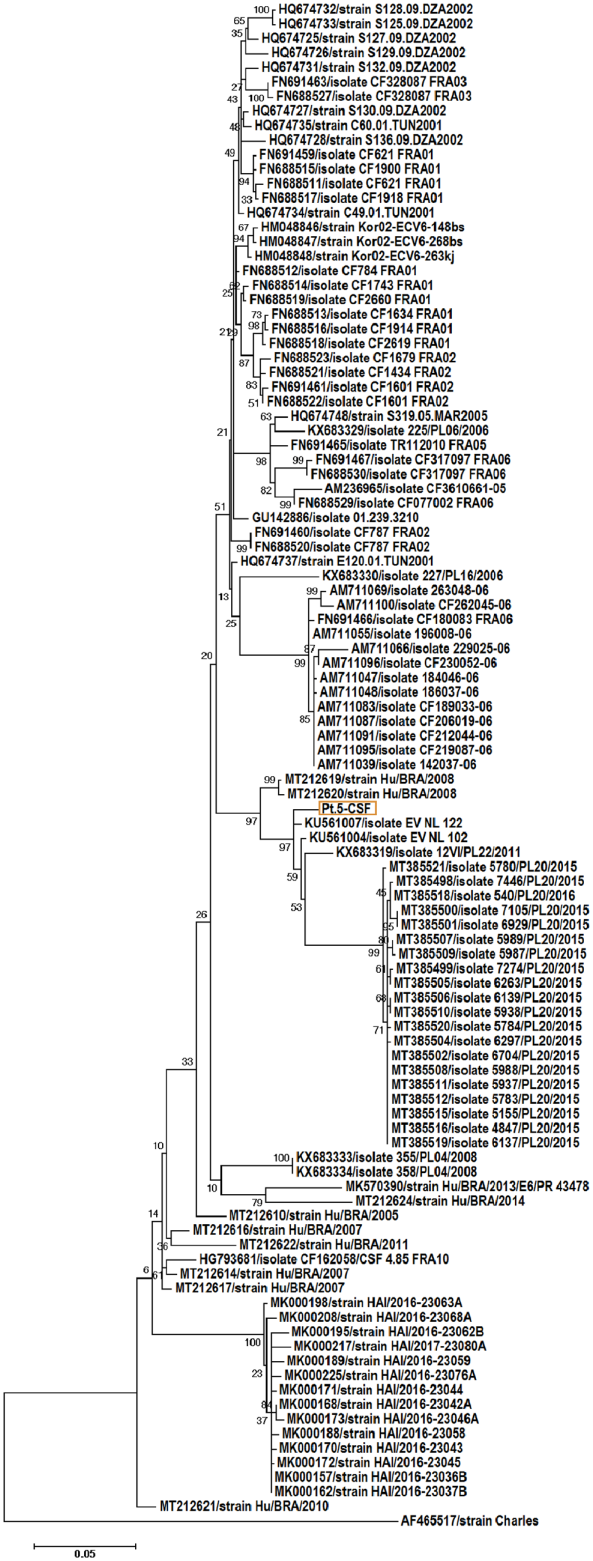
Phylogenetic tree depicting the relationships between VP1 coding region of human echovirus 6 strain isolated from CSF of Pt.5 and human echovirus 6 strains filtered from GenBank (deposited between 2010 and 2016). Each strain is referenced by its accession number. The tree was constructed by the neighbor-joining method and evaluated with 1000 bootstrap pseudoreplicates. Genetic distances were calculated with Kimura 2-parameter algorithm. Analyses were conducted using MEGA 11

The presence of enteroviral RNA in CSF was confirmed with specific PCR, and the resulting EV-VP1 product was sequenced for EV serotyping. Subsequent BLAST search confirmed the presence of human echovirus 6 – representative of enterovirus B (Fig. 2).

Our mMGS analysis in this patient detected also sequences mapping to genomes of other *Picornaviridae* including enterovirus J, *Enterovirus* sp. isolate CPML 8109/08 and porcine enterovirus 9 strain UKG/410/73, but these did not meet our criteria for mNGS identification (described in Methods).

Representatives of *Papillomaviridae* were detected in two patients, i.e., human papillomavirus type 5 (HPV5) in Pt.4 and Pt.5 and Colobus guereza papillomavirus 2 in Pt.5. In addition, mNGS analysis detected 12 reads mapping to Merkel cell polyomavirus (MCPyV) in patient Pt.10 and four unique reads mapped to human pegivirus (HPgV) in Pt.14. However, the presence of all these sequences could not be confirmed by PCR and RT-PCR. Unexpectedly, HSV was identified by DNA-mNGS in only one (Pt.18) out of 20 patients, based on two unique reads.

## Discussion

In the present study, we used mNGS to search for coexisting viral infections in 20 patients with confirmed diagnosis of HSE and detected another CNS infection in five patients. However, only in one (Pt.5), this infection (human echovirus 6) was confirmed by subsequent RT-PCR. The course of disease in this patient was uneventful, and he was discharged without any neurological sequelae.

Reports of CNS infection involving multiple pathogens are rare in the literature and usually involve viruses from the *Herpesviridae* family. In one study of CSF samples collected from patients with various neurological diseases, including viral meningitis, a mixed infection of HSV1 and HSV2 was found in 36.6% (Taj and Jamil 2018), whereas in another study on a similar group of patients, it was only 1.3% (Shikova et al. 2022). Mixed CNS infections with HSV2 and cytomegalovirus (CMV) were reported in patients with acquired immunodeficiency syndrome (AIDS) (Laskin et al. 1987; Zahid et al. 2021) and in those receiving chemoradiotherapy (Suzuki et al. 2008), but also in patients without any known immune deficiency (Xue et al. 2015).

Weinberg et al. described 16 patients with CNS coinfection by Epstein-Barr virus (EBV) and at last one other pathogen. Among 10 immunocompromised patients, three were coinfected by CMV, two by JC virus, and two by varicella zoster virus (VZV). Moreover, in three of these patients, non-viral pathogens were detected in the CSF (two were infected with *pneumococcus* and one was infected with *Cryptococcus* species). In the immunocompetent group of six patients, three were coinfected with another virus (HSV, varicella zoster virus; VZV, West Nile virus; WNV), while two were coinfected with *Ehrlichia chaffeensis* and one with *Mycoplasma pneumoniae* (Weinberg et al. 2005). The number of coinfecting pathogens could be occasionally sizeable; Kumar et al. described two unusual cases of encephalitis in 6- and 7-year-old girls from India in whom six different pathogens: JEV, Dengue virus (DENV), Chikungunya virus (CHIKV), CMV, HSV2, and Rubella virus (RuV) were detected in CSF (Kumar et al. 2019, 2020).

It is likely that immunosuppression could facilitate coinfection by multiple pathogens. VanderVeen et al. reported the case of a 59-year-old man without preexisting immune deficiencies who developed encephalitis caused by a coinfection with VZV and Jamestown canyon virus (JCV) after treatment with corticosteroids (VanderVeen et al. 2020).

In the present study, one out of 20 HSV1-infected patients was found to be coinfected with human echovirus 6. While there are few reports on coinfection with bacteria including *Streptococcus pneumoniae*, *Mycobacterium tuberculosis*, and *Neisseria meningitides* (Basmaci et al. 2011; Pelkonen et al. 2012) and viral agents like mumps virus (two cases; 3% of 66 patients with aseptic meningitis (Rasti et al. 2016) or EBV (one case; 2% of 49 patients with lymphomonocytary meningitis) (Vidal et al. 2011), EV and HSV coinfection in the setting of neuroinfection has not been described previously. This is surprising since EVs (25%) and HSV (24%) were the two most common causes of viral encephalitis in the large California Encephalitis Project (Glaser et al. 2006), while in Poland, 24% and 6.3% of encephalitis cases were found to be due to HSV and EVs, respectively (Lipowski et al. 2017). In a Polish study of children hospitalized with CNS infection attributed to enteroviruses, 6% of all patients were infected by human echovirus 6 (Toczylowski et al. 2020).

Not unexpectedly, sequences of MCPyV, HPV5 and HPgV identified in mNGS were not confirmed by specific PCRs. MCPyV and HPV5 sequences are common external contaminants which could originate from laboratory surfaces (Foulongne et al. 2011) and patient/technician’s skin (Harwood et al. 2004), respectively. Similarly, detection of HPgV sequences could be due to laboratory contamination since our lab performed extensive HPgV amplification and sequencing in the past (Bukowska-Osko et al. 2018; Kisiel et al. 2013). Much like HPgV, our mNGS runs occasionally detect human immunodeficiency virus (HIV) and hepatitis C virus (HCV) sequences which are considered our lab background noise.

Surprisingly, while detected reads pointed to EV A and EV B coinfection, serotyping confirmed infection with EV B species only. Thus, while mNGS detected the virus, it was inaccurate with regard to specific serotype. It is possible that EV A reads were in fact EV B sequences altered by PCR and/or sequencing errors and were more efficiently aligned to the wrong genome. All detected EV A reads were aligning to the same region of viral genome as the majority of identified EV B sequences (5′ untranslated region; 5′-UTR), thus giving credence to this explanation.

The problem of polymerase errors in metagenomics studies is well known and is most pronounced for whole genome amplification (WGA) kits — such as the one used in the current study — as these do not contain high fidelity enzymes (Quail et al. 2011). The errors could have also been introduced by preamplification, which was necessary as the amount of DNA/RNA in CSF was very low and similar to non-template controls (NTC); (Lauder et al. 2016), as well as by increasing the number of PCR cycles needed to generate sufficient amount of indexed DNA for sequencing during NGS library preparation (Quail et al. 2011; Sze and Schloss 2019). Errors could also occur at different steps of sequencing (clustering, cycles of sequencing, image analysis) generating mistakes in base calling ranging from 0.1 to 1% (Fox et al. 2014).

Unexpectedly, we have detected HSV in only one out of 20 HSE PCR-positive patients, indicating the possible problem of sensitivity of our mNGS procedure compared to PCR assays (Perlejewski et al. 2020). In the diagnostic center where mNGS was used in clinical settings, approx. 29% of metagenomic results matched with outputs of routine diagnostics conducted on various samples including CSF, blood, and stool (Kufner et al. 2019). Lower compatibility (42%) between metagenomics and routine diagnostic tests was shown in the study where different causative agents of CNS infection (including bacteria and fungi) were detected (Wilson et al. 2019). On the other hand, Si et al. detected HSV-DNA in CSF of all out of nine patients with suspected encephalitis, however, in five subjects number of viral unique reads was ≤ 2 (Si et al. 2023). Another study confirmed that mNGS-based pipeline may demonstrate analytical and clinical performance comparable to that of qPCR when used to detect transplant-related DNA viruses in plasma (Carpenter et al. 2019).

In our study, sample degradation was not an issue of lower mNGS sensitivity as all samples were HSV1-positive at the time of the study when tested by an in-house assay. Low sensitivity of our mNGS protocol could have been further compromised by DNase treatment which is a commonly used technique to lower the DNA background — viral genomic DNA is assumed to remain protected from this nuclease by the presence of viral envelope (Hall et al. 2014). However, herpesviral DNA in such clinical samples as serum and plasma was reported to be highly fragmented and thus susceptible to DNases (Boom et al. 2002). There may be a trade-off: DNase-free conditions in mNGS protocols may result in higher number of viral reads for herpesviruses but at the same time lower the number of sequences for non-herpes DNA and RNA viruses (Edridge et al. 2019).

In conclusion, employing metagenomic analysis of CSF from 20 patients with HSE, we identified a coexisting infection with echovirus 6 in one. While mNGS allows for the detection of a wide spectrum of viruses, contamination issues and false signals produced during analysis may complicate its clinical usefulness. Despite that, mNGS has repeatedly proven to be a powerful diagnostic tool in viral detection (Carpenter et al. 2019; Lipowski et al. 2017; Si et al. 2023).

## Data Availability

The data generated during and analyzed during the current study are available from the corresponding author on reasonable request.

## References

[CR1] Bankevich A, Nurk S, Antipov D, Gurevich AA, Dvorkin M, Kulikov AS, Lesin VM, Nikolenko SI, Pham S, Prjibelski AD, Pyshkin AV, Sirotkin AV, Vyahhi N, Tesler G, Alekseyev MA, Pevzner PA (2012). SPAdes: a new genome assembly algorithm and its applications to single-cell sequencing. J Comput Biol.

[CR2] Basmaci R, Mariani P, Delacroix G, Azib S, Faye A, Taha MK, Bingen E, Bonacorsi S, Romero JR, Rotbart HA, Nyquist AC, Nolte FS (2011). Enteroviral meningitis does not exclude concurrent bacterial meningitis. J Clin Microbiol.

[CR3] Berkhout RJ, Tieben LM, Smits HL, Bavinck JN, Vermeer BJ, ter Schegget J (1995). Nested PCR approach for detection and typing of epidermodysplasia verruciformis-associated human papillomavirus types in cutaneous cancers from renal transplant recipients. J Clin Microbiol.

[CR4] Bolger AM, Lohse M, Usadel B (2014). Trimmomatic: a flexible trimmer for Illumina sequence data. Bioinformatics.

[CR5] Bookstaver PB, Mohorn PL, Shah A, Tesh LD, Quidley AM, Kothari R, Bland CM, Weissman S (2017). Management of viral central nervous system infections: a primer for clinicians. J Cent Nerv Syst Dis.

[CR6] Boom R, Sol CJA, Schuurman T, van Breda A, Weel JFL, Beld M, ten Berge IJM, Wertheim-van Dillen PME, de Jong MD (2002). Human cytomegalovirus DNA in plasma and serum specimens of renal transplant recipients is highly fragmented. J Clin Microbiol.

[CR7] Bukowska-Osko I, Perlejewski K, Pawelczyk A, Rydzanicz M, Pollak A, Popiel M, Cortes KC, Paciorek M, Horban A, Dzieciatkowski T, Radkowski M, Laskus T (2018). Human pegivirus in patients with encephalitis of unclear etiology, Poland. Emerg Infect Dis.

[CR8] Bukowska-Osko I, Perlejewski K, Nakamura S, Motooka D, Stokowy T, Kosinska J, Popiel M, Ploski R, Horban A, Lipowski D, Caraballo Cortes K, Pawelczyk A, Demkow U, Stepien A, Radkowski M, Laskus T (2016) Sensitivity of next-generation sequencing metagenomic analysis for detection of RNA and DNA viruses in cerebrospinal fluid: the confounding effect of background contamination. Adv Exp Med Biol 10.1007/5584_2016_42 27405447

[CR9] Carpenter ML, Tan SK, Watson T, Bacher R, Nagesh V, Watts A, Bentley G, Weber J, Huang C, Sahoo MK, Hinterwirth A, Doan T, Carter T, Dong Q, Gourguechon S, Harness E, Kermes S, Radhakrishnan S, Wang G, Quiroz-Zarate A, Ching J, Pinsky BA (2019) Metagenomic next-generation sequencing for identification and quantitation of transplant-related DNA viruses. J Clin Microbiol 57 10.1128/JCM.01113-19 PMC6879295 31554674

[CR10] Chaudhuri A, Kennedy PG (2002). Diagnosis and treatment of viral encephalitis. Postgrad Med J.

[CR11] Edridge AWD, Deijs M, van Zeggeren IE, Kinsella CM, Jebbink MF, Bakker M, van de Beek D, Brouwer MC, van der Hoek L (2019) Viral metagenomics on cerebrospinal fluid. Genes (Basel) 10 10.3390/genes10050332 PMC6562652 31052348

[CR12] Foulongne V, Courgnaud V, Champeau W, Segondy M (2011). Detection of Merkel cell polyomavirus on environmental surfaces. J Med Virol.

[CR13] Fox EJ, Reid-Bayliss KS, Emond MJ, Loeb LA (2014) Accuracy of next generation sequencing platforms. Next Gener Seq Appl 1 10.4172/jngsa.1000106 PMC4331009 25699289

[CR14] Glaser CA, Honarmand S, Anderson LJ, Schnurr DP, Forghani B, Cossen CK, Schuster FL, Christie LJ, Tureen JH (2006). Beyond viruses: clinical profiles and etiologies associated with encephalitis. Clin Infect Dis.

[CR15] Hall RJ, Wang J, Todd AK, Bissielo AB, Yen SH, Strydom H, Moore NE, Ren XY, Huang QS, Carter PE, Peacey M (2014). Evaluation of rapid and simple techniques for the enrichment of viruses prior to metagenomic virus discovery. J Virol Methods.

[CR16] Harwood CA, Surentheran T, Sasieni P, Proby CM, Bordea C, Leigh IM, Wojnarowska F, Breuer J, McGregor JM (2004). Increased risk of skin cancer associated with the presence of epidermodysplasia verruciformis human papillomavirus types in normal skin. Br J Dermatol.

[CR17] Hinson VK, Tyor WR (2001). Update on viral encephalitis. Curr Opin Neurol.

[CR18] Katano H, Ito H, Suzuki Y, Nakamura T, Sato Y, Tsuji T, Matsuo K, Nakagawa H, Sata T (2009). Detection of Merkel cell polyomavirus in Merkel cell carcinoma and Kaposi's sarcoma. J Med Virol.

[CR19] Kennedy PGE (2004). Viral encephalitis: causes, differential diagnosis, and management. J Neurol Neurosurg Psychiatry.

[CR20] Kennedy PGE, Quan PL, Lipkin WI (2017) Viral encephalitis of unknown cause: current perspective and recent advances. Viruses-Basel 9 10.3390/v9060138 PMC5490815 28587310

[CR21] Kisiel E, Cortez KC, Pawelczyk A, Osko IB, Kubisa N, Laskus T, Radkowski M (2013). Hepatitis G virus/GBV-C in serum, peripheral blood mononuclear cells and bone marrow in patients with hematological malignancies. Infect Genet Evol.

[CR22] Kufner V, Plate A, Schmutz S, Braun DL, Gunthard HF, Capaul R, Zbinden A, Mueller NJ, Trkola A, Huber M (2019) Two years of viral metagenomics in a tertiary diagnostics unit: evaluation of the first 105 cases. Genes (Basel) 10 10.3390/genes10090661 PMC6770117 31470675

[CR23] Kumar M, Topno RK, Madhukar M, Pandey K, Mishra B, Sahoo GC, Singh A, Kamble B, Das P (2019) Acute encephalitis syndrome child patient with multi-viral co-infection: a rare case report. J Med Allied Sci

[CR24] Kumar M, Topno RK, Singh BK, Madhukar M, Kamble B, Sahoo GC, Das P, Pandey K, Singh A (2020). Multiple viral co-infections in a pediatric patient of acute encephalitis syndrome (AES) - an unique case report. Int J Trop Dis Health 22–27

[CR25] Kupila L, Vuorinen T, Vainionpaa R, Hukkanen V, Marttila RJ, Kotilainen P (2006). Etiology of aseptic meningitis and encephalitis in an adult population. Neurology.

[CR26] Langmead B, Salzberg SL (2012). Fast gapped-read alignment with Bowtie 2. Nat Methods.

[CR27] Laskin OL, Stahl-Bayliss CM, Morgello S (1987). Concomitant herpes simplex virus type 1 and cytomegalovirus ventriculoencephalitis in acquired immunodeficiency syndrome. Arch Neurol.

[CR28] Lauder AP, Roche AM, Sherrill-Mix S, Bailey A, Laughlin AL, Bittinger K, Leite R, Elovitz MA, Parry S, Bushman FD (2016). Comparison of placenta samples with contamination controls does not provide evidence for a distinct placenta microbiota. Microbiome.

[CR29] Leitch EC, Harvala H, Robertson I, Ubillos I, Templeton K, Simmonds P (2009). Direct identification of human enterovirus serotypes in cerebrospinal fluid by amplification and sequencing of the VP1 region. J Clin Virol.

[CR30] Les K, Przybylski M, Dzieciatkowski T, Mlynarczyk G (2010). Detection of human enteroviruses with real-time PCR assay using TaqMan fluorescent probe. Med Dosw Mikrobiol.

[CR31] Li H, Handsaker B, Wysoker A, Fennell T, Ruan J, Homer N, Marth G, Abecasis G, Durbin R, Genome Project Data Processing S (2009). The sequence alignment/map format and SAMtools. Bioinformatics.

[CR32] Lipowski D, Popiel M, Perlejewski K, Nakamura S, Bukowska-Osko I, Rzadkiewicz E, Dzieciatkowski T, Milecka A, Wenski W, Ciszek M, Debska-Slizien A, Ignacak E, Cortes KC, Pawelczyk A, Horban A, Radkowski M, Laskus T (2017). A cluster of fatal tick-borne encephalitis virus infection in organ transplant setting. J Infect Dis.

[CR33] Lunter G, Goodson M (2011). Stampy: a statistical algorithm for sensitive and fast mapping of Illumina sequence reads. Genome Res.

[CR34] Machura P, Gorka E, Mlynarczyk-Bonikowska B, Majewska A, Malejczyk M, Mlynarczyk G, Dzieciatkowski T (2015). Novel multiplex real-time PCR assay for detection and differentiation of herpes simplex virus type 1 and 2 DNA. Med Dosw Mikrobiol.

[CR35] McMurdie PJ, Holmes S (2013). phyloseq: an R package for reproducible interactive analysis and graphics of microbiome census data. PLoS ONE.

[CR36] Pelkonen T, Roine I, Anjos E, Kaijalainen S, Roivainen M, Peltola H, Pitkaranta A (2012). Picornaviruses in cerebrospinal fluid of children with meningitis in Luanda, Angola. J Med Virol.

[CR37] Perlejewski K, Popiel M, Laskus T, Nakamura S, Motooka D, Stokowy T, Lipowski D, Pollak A, Lechowicz U, Cortes KC, Stpien A, Radkowski M, Bukowska-Osko I (2015). Next-generation sequencing (NGS) in the identification of encephalitis-causing viruses: Unexpected detection of human herpesvirus 1 while searching for RNA pathogens. J Virol Methods.

[CR38] Perlejewski K, Bukowska-Osko I, Rydzanicz M, Pawelczyk A, Caraballo Corts K, Osuch S, Paciorek M, Dzieciatkowski T, Radkowski M, Laskus T (2020). Next-generation sequencing in the diagnosis of viral encephalitis: sensitivity and clinical limitations. Sci Rep.

[CR39] Popiel M, Perlejewski K, Bednarska A, Dzieciatkowski T, Paciorek M, Lipowski D, Jablonowska M, Czeszko-Paprocka H, Bukowska-Osko I, Cortes KC, Pawelczyk A, Fic M, Horban A, Radkowski M, Laskus T (2017) Viral etiologies in adult patients with encephalitis in Poland: a prospective single center study. Plos One 12 10.1371/journal.pone.0178481 PMC5453691 28570620

[CR40] Quail MA, Otto TD, Gu Y, Harris SR, Skelly TF, McQuillan JA, Swerdlow HP, Oyola SO (2011). Optimal enzymes for amplifying sequencing libraries. Nat Methods.

[CR41] Radkowski M, Wang LF, Cianciara J, Rakela J, Laskus T (1999). Analysis of hepatitis G virus/GB virus C quasispecies and replication sites in human subjects. Biochem Biophys Res Commun.

[CR42] Radmard S, Reid S, Ciryam P, Boubour A, Ho N, Zucker J, Sayre D, Greendyke WG, Miko BA, Pereira MR, Whittier S, Greens DA, Thakur KT (2019) Clinical utilization of the FilmArray meningitis/encephalitis (ME) multiplex polymerase chain reaction (PCR) Assay. Front Neurol 10 10.3389/fneur.2019.00281 PMC6443843 30972012

[CR43] Rasti M, Makvandi M, Neisi N, Azaran A, Rastegarvand N, Khalafkhany D, Jahangirnezhad E, Teimoori A, Hadian M, Shabani A, Shamsizadeh A, Nikfar R, Varnaseri M (2016). Three cases of mumps virus and enterovirus coinfection in children with enteroviral meningitis. Medicine (baltimore).

[CR44] Salimi H, Cain MD, Klein RS (2016). Encephalitic arboviruses: emergence, clinical presentation, and neuropathogenesis. Neurotherapeutics.

[CR45] Schlaberg R, Chiu CY, Miller S, Procop GW, Weinstock G, Professional Practice C, Committee on Laboratory Practices of the American Society for M, Microbiology Resource Committee of the College of American P (2017). Validation of metagenomic next-generation sequencing tests for universal pathogen detection. Arch Pathol Lab Med.

[CR46] Schulze MH, Volker FM, Lugert R, Cooper P, Hasenclever K, Gross U, Pfister H, Silling S (2016). High prevalence of human papillomaviruses in Ghanaian pregnant women. Med Microbiol Immunol.

[CR47] Shikova E, Alexandrova D, Kumanova A, Tarnev I, Vassileva E, Pacheva I, Galabova F, Pishmisheva M (2022) Herpes simplex virus infection in Bulgarian patients with neurological diseases. J Clinl Virol Plus 2

[CR48] Si Z, Li L, Han J (2023). Analysis of metagenomic next-generation sequencing (mNGS) in the diagnosis of herpes simplex virus (HSV) encephalitis with normal cerebrospinal fluid (CSF). Infect Drug Resist.

[CR49] Suzuki HI, Hangaishi A, Hosoya N, Watanabe T, Kanda Y, Motokura T, Chiba S, Kurokawa M (2008). Herpes simplex encephalitis and subsequent cytomegalovirus encephalitis after chemoradiotherapy for central nervous system lymphoma: a case report and literature review. Int J Hematol.

[CR50] Sze MA, Schloss PD (2019) The impact of DNA polymerase and number of rounds of amplification in PCR on 16S rRNA gene sequence data. mSphere 4 10.1128/mSphere.00163-19 PMC6531881 31118299

[CR51] Taj A, Jamil N (2018) Co-occurrence of herpes simplex virus 1 and 2 in patients suspected with different neurological ailments in Karachi

[CR52] Tan le V, van Doorn HR, Nghia HD, Chau TT, Tu le TP, de Vries M, Canuti M, Deijs M, Jebbink MF, Baker S, Bryant JE, Tham NT, NT BK, Boni MF, Loi TQ, Phuong le T, Verhoeven JT, Crusat M, Jeeninga RE, Schultsz C, Chau NV, Hien TT, van der Hoek L, Farrar J, de Jong MD (2013) Identification of a new cyclovirus in cerebrospinal fluid of patients with acute central nervous system infections. mBio 4:e00231–13 10.1128/mBio.00231-13 PMC3684831 23781068

[CR53] Toczylowski K, Wieczorek M, Bojkiewicz E, Wietlicka-Piszcz M, Gad B, Sulik A (2020) Pediatric enteroviral central nervous system infections in Bialystok, Poland: epidemiology, viral types, and drivers of seasonal variation. Viruses 12 10.3390/v12080893 PMC7472221 32824117

[CR54] Tunkel AR, Glaser CA, Bloch KC, Sejvar JJ, Marra CM, Roos KL, Hartman BJ, Kaplan SL, Scheld WM, Whitley RJ, Infectious Diseases Society of A (2008). The management of encephalitis: clinical practice guidelines by the Infectious Diseases Society of America. Clin Infect Dis.

[CR55] VanderVeen N, Nguyen N, Hoang K, Parviz J, Khan T, Zhen A, Jagger BW (2020). Encephalitis with coinfection by Jamestown canyon virus (JCV) and varicella zoster virus (VZV). Idcases.

[CR56] Venkatesan A, Geocadin RG (2014). Diagnosis and management of acute encephalitis: a practical approach. Neurol Clin Pract.

[CR57] Vidal LRR, de Almeida SM, de Messias-Reason IJ, Nogueira MB, Debur MD, Pessa LFC, Pereira LA, Rotta I, Takahashi GRD, da Silveira CS, Araujo JMR, Raboni SM (2011). Enterovirus and herpesviridae family as etiologic agents of lymphomonocytary meningitis, Southern Brazil. Arq Neuropsiquiatr.

[CR58] Weinberg A, Bloch KC, Li S, Tang YW, Palmer M, Tyler KL (2005). Dual infections of the central nervous system with Epstein-Barr virus. J Infect Dis.

[CR59] WHO (2004) Polio Laboratory Manual, 4th ed. Geneva, Switzerland. https://apps.who.int/iris/handle/10665/68762

[CR60] Wilson MR, Naccache SN, Samayoa E, Biagtan M, Bashir H, Yu G, Salamat SM, Somasekar S, Federman S, Miller S, Sokolic R, Garabedian E, Candotti F, Buckley RH, Reed KD, Meyer TL, Seroogy CM, Galloway R, Henderson SL, Gern JE, DeRisi JL, Chiu CY (2014). Actionable diagnosis of neuroleptospirosis by next-generation sequencing. N Engl J Med.

[CR61] Wilson MR, Sample HA, Zorn KC, Arevalo S, Yu G, Neuhaus J, Federman S, Stryke D, Briggs B, Langelier C, Berger A, Douglas V, Josephson SA, Chow FC, Fulton BD, DeRisi JL, Gelfand JM, Naccache SN, Bender J, Dien Bard J, Murkey J, Carlson M, Vespa PM, Vijayan T, Allyn PR, Campeau S, Humphries RM, Klausner JD, Ganzon CD, Memar F, Ocampo NA, Zimmermann LL, Cohen SH, Polage CR, DeBiasi RL, Haller B, Dallas R, Maron G, Hayden R, Messacar K, Dominguez SR, Miller S, Chiu CY (2019). Clinical metagenomic sequencing for diagnosis of meningitis and encephalitis. N Engl J Med.

[CR62] Xue C, Chen S, Lin Q, Zhou H, Huang C, Lin J, Xie W, Chen K, Zhou D, Ma W, Ma F, Xu H (2015). Double encephalitis with herpes simplex virus type II and cytomegalovirus in an elder Chinese: a case report. Neuropsychiatr Dis Treat.

[CR63] Zahid M, Kumar K, Patel H (2021) Encephalitis due to co-infection with cytomegalovirus and herpes simplex virus type 2 in a patient with acquired immunodeficiency syndrome. Am J Case Rep 22 10.12659/AJCR.931821 PMC8351248 34349095

